# Spirituality in Renal Supportive Care: A Thematic Review

**DOI:** 10.3390/healthcare3041174

**Published:** 2015-11-16

**Authors:** Richard Egan, Sarah Wood, Rod MacLeod, Robert Walker

**Affiliations:** 1 Preventive and Social Medicine, Dunedin School of Medicine, University of Otago, PO Box 56, Dunedin 9054, New Zealand; 2 Department of Preventive & Social Medicine, Dunedin School of Medicine, University of Otago, Dunedin 9054, New Zealand; E-Mail: sarah.wood@otago.ac.nz; 3 Hammond Care and Northern Clinical School, University of Sydney, Sydney 2065, Australia; E-Mail: rmacleod@hammond.com.au; 4 Department of Medicine, Dunedin School of Medicine, University of Otago, Dunedin 9054, New Zealand; E-Mail: rob.walker@otago.ac.nz

**Keywords:** renal supportive care, end-of-life, spirituality, religion, palliative care, chronic kidney disease

## Abstract

Chronic kidney disease is marked by a reduced life expectancy and a high symptom burden. For those who reach end-stage renal disease, the prognosis is poor, and this combined with the growing prevalence of the disease necessitates supportive and palliative care programmes that will address people’s psychosocial, cultural and spiritual needs. While there is variation between countries, research reveals that many renal specialist nurses and doctors are reluctant to address spirituality, initiate end-of-life conversations or implement conservative treatment plans early. Yet, other studies indicate that the provision of palliative care services, which includes the spiritual dimension, can reduce symptom burden, assist patients in making advanced directives/plans and improve health-related quality of life. This review brings together the current literature related to renal supportive care and spirituality under the following sections and themes. The introduction and background sections situate spirituality in both healthcare generally and chronic kidney disease. Gaps in the provision of chronic kidney disease spiritual care are then considered, followed by a discussion of the palliative care model related to chronic kidney disease and spirituality. Chronic kidney disease spiritual needs and care approaches are discussed with reference to advanced care planning, hope, grief and relationships. A particular focus on quality of life is developed, with spirituality named as a key dimension. Finally, further challenges, such as culture, training and limitations, are explicated.

## 1. Introduction

In many developed countries, spirituality is increasingly considered in healthcare policy [1,2], curricula [3,4] and delivery [5]. While tensions and controversies exist around the role of spirituality [6,7] in healthcare, research has reached the stage where the association with other health outcomes is strong enough to justify its inclusion [8]. As a result of an international consensus process, Puchalski *et al.* [9] offer a broad definition of spirituality that allows for an inclusive approach:

“*Spirituality is a dynamic and intrinsic aspect of humanity through which persons seek ultimate meaning, purpose, and transcendence, and experience relationship to self, family, others, community, society, nature, and the significant or sacred. Spirituality is expressed through beliefs, values, traditions, and practices.*”

This eclectic “map of the terrain” [10] has become, for most, the accepted approach to spirituality in healthcare [11] with hospice and palliative care, alongside an indigenous voice [12,13] leading much of the research and developments [14] in this area. It is in this context that renal care appears to be coming to terms with the importance of spirituality [15].

This review begins by situating spirituality in both healthcare generally and chronic kidney disease (CKD). Gaps in the provision of CKD spiritual care are then considered, followed by a discussion of the palliative care model related to CKD and spirituality. CKD spiritual needs and care approaches are discussed with reference to advanced care planning, hope, grief and relationships. A particular focus on quality of life is developed, with spirituality named as a key dimension. Finally, further challenges, such as culture, training and limitations, are explicated.

## 2. Search Strategy

The overall theme of the review was spirituality in CKD supportive care. The authors began their search with some obvious themes in mind, such as “gaps in spirituality”, “spiritual care needs” and “palliative care needs,” while other themes, such as “quality of life” and “spiritual care in grief and bereavement,” came from a deductive approach to the literature [16]. There was a paucity of papers that explicitly addressed spirituality and CKD as their primary focus; therefore, papers were included if they made reference to spirituality as it related to models and approaches, such as palliative care, advanced care planning and quality of life. Multi-field database searches were conducted using Medline, Scopus, PubMed, CINAHL (Cumulative Index to Nursing and Allied Health Literature), Ovid and Google Scholar. The search terms included a truncated form of spirituality combined with renal, kidney, palliative care and end-of-life care. Manual searches of original and review articles were also conducted. A further search was conducted of the policies and guidelines of equivalently-developed English speaking countries. The search was largely limited to studies between 2000 and 2015 and those published in English.

## 3. Background to Spirituality in Chronic Kidney Disease

Spirituality is ubiquitously cited as a core component of palliative and supportive care in CKD [17,18,19,20]. The World Health Organisation recognises the vital role spirituality plays in complex and chronic illnesses [21,22], such as CKD can be described. However, there remains a dearth of evidence and models to illustrate how best to incorporate spirituality into quality supportive care plans for patients with CKD and their families. Davison [23] expresses the need for more comprehensive research into alleviating spiritual suffering in the CKD population. Holley’s [24] projection of goals of care for CKD patients asks specialists to look beyond treatment pathways to encompass the psychosocial and spiritual aspects of care, such as quality of life and hope; a sentiment echoed by other researchers [25]. International frameworks, guidelines and policies similarly accord with this holistic approach to supportive care in CKD [17,25,26].

Researchers contend that patients with chronic and debilitating illnesses are prompted to examine existential and spiritual issues as a means of adjusting to their illness and coping with the omnipresence of death [27,28,29]. Breitbart [30] delves further and suggests that end-of-life (EOL) “*symptoms relating to psychological distress and existential concerns are even more prevalent than pain and other physical symptoms.*” Patients with chronic conditions have previously indicated that they would like physicians to attend to their spiritual concerns [31,32,33], especially those who face an irreversible disease trajectory [27,34,35,36,37]. The literature further reveals discrepancies between the reported spiritual needs of patients [34,38] and the actual provision of spiritual care, suggesting there are institutional gaps in meeting these needs [23,31,38].

Religion, as a concept and lived reality, precedes the growth of spirituality in the academic health discourse [8]. While spirituality in healthcare is understood broadly [39] and many studies now focus on this broad construct [40], this is not to undermine the impact that religious spirituality has on patient outcomes. Nephrology research has addressed religion, specifically in the areas of culture and truth telling [41], religious support, involvement and coping [37,42,43,44], as well as denominational-specific studies [45,46]. These and many other studies from different disease areas [47] suggest a strong association between religion and positive health outcomes, quality of life (QOL), coping mechanisms and as a protective factor, reinforcing the need for regular religious assessment and care. There are other studies that show that religion can be negatively associated with health outcomes, particularly when related to guilt and punishment [48]. The religious/spiritual mechanisms involved, whether positive or negative, are still not clear, and many researchers call for further research [32,47,49,50]. The following discussion, while referring to spirituality, encompasses the range of spiritualties from religious to atheist.

## 4. Gaps and Challenges in the Provision of Spiritual Care in CKD

It is evident that there are gaps in the provision of spiritual care in CKD. For example, in Tanyi *et al.* [51], a small qualitative study of women undergoing haemodialysis in the United States (U.S.), some participants perceived their nurses’ reluctance to engage in spiritual or religious discussions as a hindrance to receiving spiritual care. Egan *et al.* [52] qualitative study of nephrology specialists investigated the provision of spiritual care in New Zealand (NZ) renal units and found that while nephrology specialists and nurses were empathetic to patients’ care needs, they lacked confidence and training opportunities to attend to patients’ spiritual care needs. While most respondents in Davison’s [23] Canadian study of EOL care preferences and needs of patients with CKD considered it important to include QOL, psychosocial and spiritual concerns in renal care programmes, less than 10% reported having had a discussion about EOL care with their nephrologist in the last twelve months, and the majority had poor self-reported knowledge of palliative care options. Patients in this study desired more education and support related to EOL issues for both staff, patients and families, greater family involvement in care and decision-making and regular EOL care discussions with nephrologists. Davison and Jhangri [28] in their Canadian prospective cohort study assessing the spiritual and supportive care needs of patients with CKD recorded 32% as having high spiritual needs (defined as reporting more than five of the seven needs listed), while 69.1% of patients reported having at least one need. The most prevalent spiritual needs identified by participants were finding hope and meaning, followed by attention to death and dying. Interestingly, no clear predictors of spiritual need were identified, illustrating the need to assess all patients for unmet needs from the onset of diagnosis and regularly thereafter.

### Training, Education and Other Issues

Despite the presence of national guidelines and specialist frameworks scaffolding nephrology supportive care practice, studies repeatedly signal gaps in the provision of palliative care and, hence, spiritual care, which start with EOL care training and education [17,28,36,53,54,55,56,57,58]. While varying between countries, there is some evidence that nephrologists have shown a reluctance to initiate EOL discussions [17,53,54,56,59] as suggested by late referral to hospice or palliative care [53,57], patients being unaware that they are near EOL [56] and regrets about commencing dialysis [23]. Collins and Lehane’s [60] quantitative study of CKD patients’ perspectives of death, dying and advanced care planning (ACP) found that 88% wanted honest answers from their doctor, 84% wanted comfort from a religious or spiritual figure and 100% wanted to be at peace spiritually. However, many nephrologists continue to lack palliative care training and exposure to palliative medicine during fellowship rotations, as demonstrated in Combs *et al.* [61] study of nephrology fellows, which found that the ranking of teaching quality in EOL care had not changed much in a decade when compared to the findings of an earlier study [62]. In the original study [62], less than 30% of the fellows reported receiving training related to cultural or spiritual issues in EOL care. In Davison *et al.* [36] study of U.S. nephrology fellows’ preparedness for EOL decision-making, 39% perceived themselves as very well prepared to make EOL decisions, leaving 61% not feeling very well prepared. A U.S.-based survey of nephrology fellows’ palliative care experiences found 72% had not undertaken a palliative care medicine rotation and 53% had no formal palliative care elective experience during residency [63]. It should be noted that the response rate in this latter study was poor. In NZ, the need for an active conservative care pathway to manage CKD stage 5 is increasingly recognised and is being introduced into renal services and the nephrology training programme.

Studies reveal that other factors may hamper the delivery of spiritual care in CKD. In Tanyi *et al.* [64] study of physicians and nurses, a lack of time and inattention to listening were perceived as barriers to delivering spiritual care. Nephrology nurses in Deal and Grassley’s [65] study identified the demands of working in a busy and noisy environment with its time constraints and lack of privacy as not conducive to providing spiritual care. Renal and palliative specialists in Levy *et al.* [66] paper on ways to improve the quality of care at EOL perceived that the barriers to good supportive care were language, workload issues, cultural and spiritual barriers between patients and the support team and environmental factors, like lack of space and privacy.

## 5. A Palliative Care Model for CKD Supportive Care

The World Health Organisation defines palliative care as “*an approach which improves the quality of life of patients and their families facing life-threatening illness, through the prevention and relief of suffering by means of early identification and impeccable assessment and treatment of pain and other problems, physical, psychosocial and spiritual*” [22]. As a life-limiting disease, this definition is relevant to CKD insofar as the assessment of patients’ supportive care needs is often delayed or limited in scope, thus exacerbating the potential for high symptom burden, emotional and spiritual distress, compromised QOL and late referral to palliative and hospice services [53,67,68,69]. Arulkumaran [70] is attuned to the importance of recognising that “*Where escalating care is futile, or a patient has expressed the limits of his treatment, an appropriately constructed palliative strategy will permit a more dignified death for the patient and better psychological outcomes for relatives.*” Previously, research has tended to focus on cancer-centred models of palliative care, thus perhaps overshadowing the needs of CKD patients who have been shown to experience at least similar distress levels and symptom burden as cancer patients [17,71,72]. In a recent Irish study exploring public perception and awareness of palliative care, the majority assumed that palliative care came under the domain of cancer care [73], a finding similar to that of an NZ study [74]. NZ records of diagnostic groups of hospice patients indicate that there is a large number of people with a non-cancer diagnosis (21% non-cancer diagnosis; 79% cancer diagnosis), including renal disease, not accessing hospice palliative care [75], a finding supported by Murray *et al.* [53] in their study of hospice use by the dialysis population in the U.S. In Davison’s [23] Canadian study of stage 4 and 5 CKD patients, 83.4% reported not knowing what palliative care was, 60.7% regretted their decision to start dialysis, 51.9% reported not having had a discussion about EOL preferences and 13.4% wanted there to be greater emphasis on spiritual care. Similarly, in McAdoo *et al.* [54] quantitative study of patients in the U.K. with advanced kidney disease, only 28% had had an EOL discussion in the year prior to death. Hobson *et al.* [55] national survey of the provision of specialist palliative care services for patients with end-stage renal disease (ESRD) in the U.K. found that while the majority of specialist palliative care services accepted ESRD patients, only a small number of referrals were reportedly made, and less than half of the service providers were aware of the National Service Framework for Renal Services [76]. It is well documented that palliative care support should be offered to end-stage kidney disease (ESKD) patients who decline or withdraw from dialysis [77]. Various practice guidelines and position statements are available to help navigate these renal pathways, like “*Shared Decision-Making in the Appropriate Initiation of and Withdrawal from Dialysis*” [26,78], the U.K. Renal National Service Framework [76] and the Australasian guidelines [17].

The U.S. Renal Physicians Association and American Society of Nephrology endorse clinicians instigating timely discussions with ESKD and CKD 5 pre-dialysis patients about their beliefs and options in managing their EOL needs [26]. They propose a palliative care model that offers pain and symptom control, advanced care planning and psychosocial and spiritual care for patients and their families. Such a broad and diverse range of supportive care needs requires a multi-disciplinary team that offers a marriage of renal medicine and palliative medicine [17,26,79]; a model that is not currently common practice in some renal services. Josland [68] gives us an example of a renal supportive care programme in Australia that relies on the cooperation of renal and palliative care services and includes amongst its team a director of palliative care, a director of renal services, a palliative care consultant, two renal clinical nurse consultants, a renal dietician and a renal social worker. This model and similar models are currently being considered by other units in Australia.

As suggested, spirituality is a widely-accepted component of the palliative care model, and it follows that it should be part of standard care practice in CKD supportive care [22]. Timeliness, communication, honesty, listening, compassion, inclusiveness and exploring meaning, purpose, identity, relationships and some religion needs are all commonly-cited tenets of spiritual care [17,26,39,51,64]. Tanyi *et al.* [51] phenomenological study of women in the U.S. undergoing haemodialysis offers us an example of care that highlights compassion and the importance of developing meaningful relationships within renal services. Participants wanted nurses to attend to their spiritual needs through genuine caring, listening, touch, kindness, patience and trust. Tanyi *et al.* later [64] qualitative study of physicians, nurses and physician assistants corroborates this patient-centred, intuitive approach, with the majority reporting that they attend to spiritual needs indirectly through presence and listening. All participants related spiritual care to genuinely caring for patients without judgment and encouraging them to draw on their own spiritual practices. Puchalski stresses the need for “empathic listening” [80], while Puchalski *et al.* [9] identify compassion and communication as important factors in emolliating spiritual distress. In Walton’s [81] small U.S. qualitative study of patients receiving haemodialysis, spirituality was associated with relationships, decision-making, problem-solving and reflection. It has been argued that spiritual care [52] occurs implicitly through relationships, but questions remain whether spiritual needs are missed if explicit assessment is not conducted.

## 6. Addressing Supportive and Spiritual Care Needs

### 6.1. Supportive Care Pathways and the Role of Spirituality

Increasing numbers of elderly people are presenting with CKD and multiple comorbidities, precipitating the need for formalised supportive care plans that focus on symptom control, psychosocial and spiritual concerns [17,82]. The burgeoning demand for palliative care and the subsequent shortage of expertise in this area makes it even more essential for nephrologists and associated staff to develop skills in EOL care [83,84].

The provision of supportive care for the elderly and comorbid is especially important, because “*patients, families, and nephrologists struggle with deciding when dialysis is prolonging life versus when it is merely delaying death*” [85]. This is illustrated in Hussain *et al*. [86] U.K. retrospective cohort study comparing survival, hospital admissions and palliative care access according to whether 70^+^-year-olds chose renal replacement therapy (RRT) or conservative treatment. The group receiving RRT survived longer; however, over the age of 80 and in patients with high co-morbidities, no survival advantage was demonstrated. Reduced QOL is another consideration in this aging population [87]. Conservative treatment provides an alternative holistic pathway for these vulnerable patients, with its focus on symptom management, psychosocial and spiritual support [15], as well as preparation for EOL. Wright *et al*. [88] longitudinal cohort study based in the U.S., for example, found that EOL discussions were associated with less invasive interventions and earlier hospice admission, while more aggressive medical care was associated with poorer patient QOL. Carson *et al.* [89] compared outcomes for patients with ESRD who chose either conservative or renal replacement therapy (RRT) and found that RRT patients had higher rates of hospitalisations and that those on a conservative care plan were more likely to die at home or hospice. Similarly, Hussain *et al.* [86] found that those who followed a conservative treatment plan had greater access to palliative care services; conversely, those receiving dialysis were more likely to be admitted to and die in hospital with little specialist palliative care. The Australia and New Zealand Dialysis and Transplant Registry (ANZDATA, 2014) showed that in 2013, of 575 deaths in Australia and 73 in NZ attributed to withdrawal from dialysis, “psychosocial” was the most commonly-cited reason for withdrawal, supporting the proposition that “*although dialysis is life-sustaining therapy and extends life, it may also create, increase or prolong suffering while not restoring or maintaining well being*” [87].

The overall poor prognosis of those who withdraw or choose not to initiate dialysis treatment necessitates a closer examination of the role of spirituality in palliative care; as a means of informing decision-making, relieving burden, addressing existential issues and as a reference for bereavement and grief support. Overall, the ability to identify those most vulnerable early is vital for best practice supportive care practice. These important issues are in the process of being addressed by a prospective accelerated longitudinal study investigating quantitative and qualitative aspects of decision-making and dialysis outcomes in individuals aged 65 years and over [90]. The results of this study will further inform this discussion.

### 6.2. Advanced Care Planning

At EOL, ACP is recommended across many of the world’s healthcare systems, such as the U.S., U.K., Canada and NZ. ACP is a coverall term suggesting preparation at EOL that involves all dimensions of care in “*a process of communication among patients, their families and their health-care providers addressing end-of-life care*” and is “*a mechanism to prepare for death*” [91,92]. Spital *et al.* [93] describe the development of a tool that measures the quality of dying that can be used by nephrologists to support patient care at EOL. One of the tool’s five domains is “peacefulness”, a key component of spirituality, and another is “advanced care planning”, highlighting the perceived relevance of both of these domains to EOL care. The authors go on to recommend the development of additional research and clinical measurement tools for EOL that reflect patients and their families’ values and preferences. Panel discussions conducted with patients and families of deceased patients in the U.S. found that they wanted measurements of dying to include pain control, dignity, spiritual/emotional needs and maintenance of the quality of life [93]. Other authors emphasise the importance of incorporating ACP into CKD patients’ treatment care plans [67,91] and echo Gawande’s [94] call for medical goals to focus more on patients’ needs and wants instead of medical interventions that may prove futile.

Given the disease trajectory of CKD, early and regularly-updated ACP discussions are widely recommended [57,91] “*long before critical care services are required*” [70]. Holley *et al.* [95] U.S. examination of how ACP impacts the EOL discussion (*n* = 400) found that of the 51% who had ACP discussions, 69% had not talked about withdrawal from dialysis. Kataoka-Yahiro *et al.* [91] conducted a cross-sectional survey (*n* = 50) exploring attitudes toward death and ACP with Asian Americans and Native Hawaiians. Of note, participants expressed death anxiety; 92% of participants said “*being at peace spiritually*” was important; only 40% had completed an ACP; and most indicated that they would prefer to have ACP discussions with family members rather than professionals. Spital *et al.* [93] similarly found that patients were more comfortable talking about EOL issues with family. Conversely, in Collins and Lehane’s [60] small study of patients receiving haemodialysis, participants were comfortable talking about death, but not with their family. These differences highlight the importance of incorporating patients’ individual beliefs, values and preferences into EOL care planning, of which ACP can play an important role. Bristowe *et al.* [96] U.K. qualitative study found that participants “*reported a lack of opportunity to discuss their future*” and suggested such discussions needed to be normalised. In Australia, Smith *et al.* [97] nurse-led initiative offers an integrated and coordinated model of care that recommends the use of validated assessment instruments, such as the Patient Outcome Symptom Scale (renal version (POS-S) for patients to rate their physical symptoms, psychological and spiritual needs and the development of ACP to support its practice. Ultimately, the aim of ACP is to work with the patient and family to create the conditions for as good a death as possible. To meet that goal, the literature and relevant guidelines suggest that the spiritual domain needs to be addressed. However, research spanning from the 1990s [95] and early 2000s [72] to the present [96] suggests that twenty years of ACP across such countries as the U.S., U.K. and others has been slow in changing the culture around death discussion and preparation for dying.

### 6.3. Hope as a Spiritual Source of Strength

Hope can be regarded as a spiritual source of strength [80,98,99] or a goal of care for patients facing a life-limiting disease [28]. Berzoff *et al.* [79] conclude that providing EOL care “*requires balancing hope and compassion with reality, and this is not always easy to do.*” Perhaps the biggest obstacle to achieving or maintaining hope in a clinical situation lies in the common assumption that delivering a poor prognosis to a patient or their family will erode hope [17,56,77,88]. Davison and Simpson’s [56] study offers an alternative perspective that stresses the role of honesty and communication in mediating hope with, “*participants express[ing] greater feelings of isolation and less hope when they were not able to honestly and openly discuss their hopes and fears for the future with loved ones.*” This need for honesty is affirmed by 60% of the haemodialysis patients who participated in Collins and Lehane’s [60] study who wanted to know if they only had six months or less to live. Disclosure and honesty must be balanced with the knowledge that not everyone desires full disclosure or involvement in decision-making, thus illustrating the need to tailor ACP to the individual [100]. In spite of the poor outcomes associated with CKD, ACP may still promote hope, because it invites open and honest discussion about EOL issues and assists patients in planning and setting goals [56]. Patients in Davison and Simpson’s [56] qualitative study identified hope as central to the process of ACP. Patients suggested that receiving more information earlier about EOL helped them to feel hope because they were able to experience stronger relationships with family and friends. Perceived barriers to, or points of reduced hope, as reported by patients include bereavement [98], the predominant focus on clinical aspects of care and relying on staff to initiate EOL discussions [56]. Relationships with family and friends have been perceived as vital to preserving a sense of hope for patients receiving haemodialysis, while maintaining hope and a sense of control has been viewed as helpful for patients adapting to the transitions and challenges of CKD [56,98].

### 6.4. Spiritual Care in Grief and Bereavement

Holley [24] adds further breadth to the role of spiritual care as encompassing grief and bereavement support for patients of CKD and their families, particularly when patients must cope with the deaths of other patients in the renal unit, as well as face their own mortality [52,81,101]. In her 2005 and 2007 review articles, Holley calls for greater use of palliative care approaches, such as ACP and spiritual support, to improve death planning and quality [24,102]. Fassett [77] reminds us that while grief is obviously associated with death, it can be experienced much earlier in the course of an illness. The chance to discuss decisions about end-of-life care and attend to psychosocial and spiritual issues have all been cited as important to patients who are nearing death. Chibnall *et al.* [103] cross-sectional study of patients with life-threatening medical conditions (*n* = 70), including kidney disease, found that death-related anxiety and depression were associated with more acute physical symptoms, greater depressive symptoms and less spiritual wellbeing and communication with physicians. Such effects on mind and body lends weight to a palliative care model that is inclusive of patients’ grief and bereavement needs as endorsed by The Renal Physicians Association and American Society of Nephrology. Family carers’ experience of coping with their loved one’s declining condition is marked by uncertainty, anxiety, fear and an avoidance to talk about death with the patient [104]. Noble *et al.* propose instigating ACP discussions with carers to help them adjust to their loved one’s disease progression and inevitable death. Some countries have developed policy and EOL plans, like The Liverpool Plan, to help facilitate a “good death” and reconcile both spiritual and cultural preferences at EOL [59], though the implementation of such approaches remains inconsistent. Holley’s [102] review of bereavement care acknowledges the role of dialysis staff who continue to provide psychosocial and spiritual support for family and loved ones after patients have died.

### 6.5. Fostering Relationships for Spiritual Care

The time commitment of those attending dialysis clinics and the chronic nature of the disease places nephrologists and staff in the unique situation of developing long-term, trusting relationships with patients [51,52,65]. For example, the majority of patients in NZ dialyze for three or more sessions per week, 4–5 h per session [82]. These relationships may allow nephrology staff to better assist patients who experience spiritual distress or EOL issues [67]. This aligns with Puchalski *et al.* [105] claim that anyone who cares for the terminally ill should be able to provide spiritual care. The nephrologists in Berzoff *et al.* [79] focus groups considered the relationships they had with patients paramount to communication about EOL issues. It should be noted that the demanding and extended duration of CKD treatment gives rise to frustration and distress, compounding the need for supportive care in this population [89].

## 7. Quality of Life

While health-related quality of life (HRQOL) has traditionally been defined in terms of physical, emotional, social and functional domains, some researchers argue that spirituality is also an important dimension [18,106]. Spiritual, religious and personal beliefs can make a substantial difference in QOL, particularly for those who report very poor health or are at the end of their life [107]. An underlying principle of palliative care is the attainment of an acceptable quality of life for the dying person and their family [1,108,109]. Renal supportive care guidelines highlight the importance of considering QOL when exploring treatment options and management pathways [17]. For instance, the Australasian guidelines for elderly CKD patients suggest that dialysis therapies may decrease the quality of life for both patients and their carers and lead to increased hospitalization [17]. Unruh *et al.* [110] review of kidney disease HRQOL issues calls for a broad understanding of HRQOL that includes four domains: physical functional, social, psychological and global (where spirituality is named). The authors note the limitations of global HRQOL measures and note that when used clinically on a case-by-case, person-centred basis, these limitations can be overcome.

While a medical intervention may be considered successful, it may not reduce the patient’s suffering [111], which is especially true of CKD, where dialysis is accompanied by significant morbidity [72]. This becomes particularly pertinent in the elderly and comorbid ESRD population for whom dialysis may negatively impact QOL. The transition from active management of a chronic disease to palliative care should focus on “maximising the quality of life” by identifying and managing symptoms, including pain, mental health issues and awareness of spiritual needs [112]. Holley [24] stresses the need to discuss life expectancy and QOL from when dialysis is initiated and emphasises that when care shifts from treatment to supportive, wellbeing and quality of life should become the focus. In the U.S., the Renal Physicians Association reiterates the importance of explaining the burden of dialysis on quality of life for ESRD patients.

Various studies of CKD patients have highlighted the role of spirituality in QOL. Using the Spiritual Well Being Questionnaire (SWBQ), Finskelstein *et al.* [20] examined spiritual wellbeing with other QOL measures in a sample of CKD patients (*n* = 200). They found strong correlations, particularly for the existential component, between the SWBQ and the QOL measures. There was no relationship found between the SWBQ scores and measures of age, comorbidity or patient compliance. The authors ask whether, “*engaging patients in discussions about their spiritual concerns and attending to their spiritual well-being may contribute to an improvement in their quality of life and medical outcome.*” Koenig *et al.* [113] in a cross-sectional study found that religiousness and spirituality predicted greater social support, fewer depressive symptoms, better cognitive function and the means to cope with illness. Davison and Jhangri [18] suggest existential wellbeing (EWB) is an independent predictor of HRQOL and conclude that spirituality needs to be targeted alongside psychosocial needs. The authors note that they found no correlation with religious wellbeing (RWB), the other sub-scale of the SWB instrument, possibly suggesting that generic aspects of spirituality, such as measured by the EWB sub-scale, have a greater impact than religious spiritual aspects. An Australian hospital-based renal supportive care programme used performance measures to evaluate the service, including the use of a validated tool to assess improvement in patients’ quality of life [68]. The hospital had measured dialysis patients’ QOL since 2001, and the researchers noted that QOL has remained persistently low compared to the normal population despite the many changes that might have improved the dialysis patients’ QOL, prompting the development of a renal supportive care programme. Kimmel *et al.* [114] in their ESRD patient QOL study interviewed U.S. haemodialysis patients (*n* = 165), using a range of instruments, including the Spiritual Belief Scale and McGill QOL scale, and concluded that spirituality was important for ESRD patients’ quality of life; however, there was no direct correlation with clinical parameters. Weisbord *et al.* [72] undertook a small study that explored QOL, ACP and palliative care. Even after an intervention from palliative care specialists, the patients did not increase ACP use, and there was no measurable change in symptom burden or HRQOL; however, participants were generally positive about the meetings. There appear to be few specific renal HRQOL tools available, and some measures of HRQOL lack the spiritual domain [18].

## 8. New Zealand Provides a Cultural Perspective of CKD and Spirituality

NZ offers a unique cultural perspective, because the principles of the Treaty of Waitangi underpin the country’s public health system and legislation for improved health outcomes and reduced disparities for Māori (NZ’s indigenous people) [115,116]. Despite these aspirations, ESRD incident rates for Māori in NZ are considerably higher than non-indigenous people [82], and renal failure deaths were three and a half times more common among Māori than non-Māori in the period of 2000–2005 [117]. In recognising such disparities and the critical need for Māori at EOL, the New Zealand Palliative Care Strategy [108] recommends a policy-led approach to palliative care services that engages Māori providers and addresses end-of-life needs in a coordinated and culturally-appropriate manner. Recent studies reveal the various barriers for Māori accessing palliative care services include: a lack of communication, difficulty navigating health literacy demands and a palliative care workforce lacking cultural competency [118,119,120]. The issue of the late referral of Māori to palliative care services identified in these studies supports the need for an integrated and equitable model of palliative care that accommodates Māori cultural values, beliefs and *wairua* (spirituality).

Central to Māori health and EOL is the notion of *wairuatanga* or spirituality [121]. In two studies exploring EOL experiences of Māori and their *whanau* (family) [119,122], participants expressed dying in spiritual terms and primarily revolving around *whanau* and *tikanga* (cultural practices). Spirituality and the associated enactment of spiritual and cultural rituals appeared to fortify families, offered hope and comfort and provided a means of coping with ongoing treatments, decision-making and the reality of a poor prognosis [122]. Moeke-Maxwell *et al.* cite Reid, who reminds us that the Māori perspective of dying and bereavement cannot be generalized, that it is “*plural, it’s diverse, it’s multiple, it’s flexible and it’s changeable.*” While these findings are focused on just one country, a recent meta-synthesis of qualitative research about end-of-life experiences of indigenous peoples in Canada, Australia, NZ and the U.S. affirms the centrality of spirituality, with the overarching theme called “preparing the spirit” [123].

## 9. Limitations

This review is limited due to the radical heterogeneity of the studies, which has led to the thematic approach above. Specifically, related to the nephrology studies, there are slightly more quantitative than qualitative studies; with the majority coming out of the U.S., followed by the U.K., Canada, Australia and NZ. There is a significant lack of indigenous studies focused on spirituality and renal care. Further, many of the studies have small numbers, are cross-sectional or, if qualitative, lack a developed theoretical base. Other limitations in this renal literature include the role of the spiritual caregiver, whether an expert (chaplain and other), nephrology experts or family members.

## 10. Conclusions

Throughout the literature review, it became apparent that there is a need to grow the evidence-base guiding spiritual and supportive care practice. For example, the Kidney Disease Improving Global Outcomes (KDIGO) conference discusses the limitations in available data regarding the benefits and potential harms of dialysis *versus* conservative care for patients living with stage 5 CKD and comorbidities and highlights the ethical difficulties of pursuing this area of research [124].

Spirituality, across the theist to atheist spectrum, is not well addressed in renal care. This spirituality and renal care review has considered the literature with a particular focus on end-of-life CKD. It has suggested that although the guidelines and studies recommend ongoing and regular spiritual assessment and care, this is often not the case for many patients. There will be a growing demand for supportive care due to the rising incidence of CKD, particularly in the older age group; therefore, it is increasingly important that nephrology experts, particularly nurses and doctors in tandem with palliative care specialists, rise to the challenge of providing holistic supportive care that explicitly considers patients’ spiritual needs. CDK is a life-limiting disease that warrants spiritual approaches to care, such as the timely implementation of ACP, consideration of HRQOL and palliative pathways to ensure better outcomes, less unnecessary interventions, earlier hospice use and better quality of life and death. These recommendations are not new; they are explicit for recent renal care guidelines; however, they need to be carefully followed, and further research is needed, particularly related to well-designed large mixed method studies about spirituality and CKD.
